# Impact of climate change on primary agriculture, water sources and food security in Western Cape, South Africa

**DOI:** 10.4102/jamba.v11i1.562

**Published:** 2019-04-17

**Authors:** Elliot M. Zwane

**Affiliations:** 1 Department of Agricultural Extension and Animal Production, Centre of Rural Community Empowerment, University of Limpopo, Sovenga, South Africa

## Abstract

Climate change is undoubtedly one of the biggest crises that humanity is facing today. There is a robust scientific consensus that human-induced climate change is occurring not only in the Western Cape but around the world. The objective of this research was to assess the impact of climate change on primary agriculture and food security. The paper is based on a literature review. A variety of literature reviews, for example, 11 government reports and 21 journal articles including experience outside Western Cape, were consulted to enrich the local experience regarding the impact of climate change on agriculture. The results indicated that many dams had low water levels (40%) during 2016/2017, which reduced crop yields including grapes. Droughts, which affected both smallholder and commercial farmers, are now a common phenomenon. Livestock production has declined over time, with small stock, the beef and dairy industry being the most affected. The paper concludes by highlighting climate adaptation and mitigation interventions and strategies for both crops and livestock production in the Western Cape. The major recommendations included scaling up on the use of organic matter to avoid burning and creating gas emissions to the atmosphere, the effective use of livestock manure and the use of appropriate and adaptable seed varieties, managing the manure of the livestock to assist in mulching to reduce water loss through evaporation and using adaptable seeds.

**Keywords:** climate change; impact; mitigation; primary agriculture; adaptation; drought.

## Introduction and background

Climate change is considered to be one of the biggest challenges that communities are facing locally and internationally (IPCC 2006). The reason why climate change is interpreted as a crisis is that it affects the very sources of human livelihood that are agriculture and the environment. Any disturbance in either agriculture or the environment affects the sources of livelihood of communities. Agriculture plays a major role in food production that feeds nations. The estimated population growth of 1.5 billion in the sub-Saharan countries will expect agriculture to feed them by 2050 (FAO 2007). The projected situation of sub-Saharan countries is that they will experience challenges of climate change (Cohen et al. 2008). However, the situation has changed, with extreme heat especially in the subtropics and changes in the rainfall pattern (Serdeczny et al. 2015). Evidence suggests that the climate change is already affecting the sub-Saharan countries. In a study documented by 350 Africa.org (n.d.), it showed eight ways in which the impact of climate change affects Africa and the sub-Saharan countries. It was confirmed that livestock such as goats and sheep were dying because of a lack of grazing, and water sources were drying up, causing a serious conflict among the users. The impact of climate change also affects South Africa.

Benhin (2006) asserted that as far as agriculture in South Africa is concerned, it is a highly sophisticated and successful sector that is made up of medium- to large-scale farms. These farms are commercially oriented, capital intensive and generally produce a surplus, which account for 90% of the value added and cover 86% of the agricultural land (NDA 2000). The availability of water has become the most limiting factor when it comes to agricultural production in South Africa, especially when one considers rainfall pattern because rainfall is unevenly distributed across the country. According to DWAF (2004), South Africa receives an annual rainfall of 450 mm, which is low when compared with the world’s average of 860 mm, and it has high levels of evaporation; on the contrary, South Africa has only 10% of its area that receives more than 750 mm, of which 50% is used for agriculture.

The Western Cape is one of the nine provinces of South Africa. It lies in the Mediterranean climate zone receiving winter rainfall as opposed to the rest of the provinces. It is located on the southern tip of the African continent between the Indian and Atlantic oceans. It is bordered by the Northern Cape and Eastern Cape provinces; the region is topographic and climatically diverse (Western Cape Municipalities 2017). The Western Cape covers an area of 129 462 km^2^. It is the fourth largest province in South Africa by surface area and also ranks fourth in population. The Western Cape is rich in agriculture and fisheries. It is an ideal place for grape cultivation. Other fruits and vegetables are also grown here. The province is divided into one metropolitan municipality, five district municipalities, and 24 local municipalities (Western Cape Municipalities 2018).

Agriculture plays an important role by providing food and earning foreign currency (Hoffman & Harrison n.d.). According to Pienaar (2017), the Western Cape is one of the provinces which export agricultural products to both other provinces and other countries in the world; different industries such as the deciduous fruit, wine and citrus are found in the Western Cape, which contribute significantly to the overall agri-economy of South Africa. It contributes 2% of the gross domestic product (GDP). Apart from crops, the Western Cape has livestock. It is estimated that the Western Cape contributes 24% of the GDP in South Africa. The total contribution of agriculture to the GDP of the Western Cape is around 4% (Hoffman & Harrison n.d.).

What motivated this study was the fact that the Western Cape used to supply the other provinces with fodder; however, the situation started to change in 2014 and 2015. The water situation started to deteriorate, leading to negative performance and strict measures of controlling irrigation water. The Western Cape Department of Agriculture organised annual symposiums and speaker after speaker spoke about the need to contain the drought by capacitating the extension advisory services.

The author being one of the speakers in one of the annual symposia noticed that the adverse effect of climate change on agriculture would have severe implications in ensuring food for the region. According to Benhin (2006), South Africa and the Western Cape contribute 50% of maize as the staple diet of the South African Development Community (SADC). The author undertook this study to establish what impact the 3-year drought has had on to the primary agriculture. Of greater interest was the fact that when the inland provinces, such as Limpopo, were affected by drought in 1981–1984 and subsequent years (Maponya & Mpandeli 2016), the Western Cape was the life blood of the nation, as it would supply livestock products, food and fruits to the rest of the provinces. However, with the changes in climate, the Western Cape Province has experienced severe drought since 2015. It is the only province that enjoys a Mediterranean climate; therefore, it was imperative for the writer to explore their strategies in dealing with their primary agriculture in the wake of climate change in order to provide lessons that could be adapted for other provinces in South Africa.

## Problem statement

There is little understanding about the concept of climate change and how this phenomenon affects smallholder farmers in the Western Cape Province. Climate change has negative impacts on crops and animals, resulting in food insecurity. Given the yearly reduced crop and livestock yields, farmers and communities in the Western Cape have been baffled by a number of questions that point to their lack of understanding of the concept of climate change and variability. The major question is the following: what strategies can be adopted to mitigate climate challenges? The impact of climate change has been reported by a number of studies, people, government departments and non-state actors.

The evidence and impacts of climate change in the Western Cape are largely highlighted in the studies by Shabangu (2017); IPCC (2007) and the Government of the Western Cape (2014). Agricultural extension services become a must in the drive to increase food production. Such services provide knowledge and advice to primary food producers and facilitate awareness on the changes that occur in the environment and how such changes affect production of food in the Western Cape. This article sought to conceptualise climate change and variability as it affects smallholder farmers in the Western Cape. The impact of climate change on water sources, livestock and crops is highlighted and mitigation strategies are recommended.

## Methodology

The paper is based on a literature review. Different search engines were used to search ‘climate change’-related articles in journals and books. Of eight government reports, four were written by consultants on behalf of the government, and 21 articles were found from journals, giving the number of required studies to undertake this research. This means that many articles were consulted and the viewpoints were collected as the basis and parameters for this paper. As indicated, general reports about the Western Cape were used as well as those that reported on challenges of climate change in the Western Cape were included: such as ‘Policy and strategic documents’ Government Policy on Climate Change of (2014), the Climate Change Response Strategy in the Western Cape (2014), the Western Cape Climate Change Response Framework (2014), Green Agri, a Policy Framework for Climate Change for response in the West Coast District (2014) and the Farmer’s Weekly of May 2017. All these documents and articles were found useful in terms of expanding the frontiers of knowledge on climate change in the Western Cape. It should be noted that there was no specific study area selected for this article. The methodology was to review the different areas and to point out whether there are policies to deal with the impact of climate change in the Western Cape.

## Findings and discussion

### Conceptualisation and contextualisation of climate change

Understanding the concept of climate change is critical because many researchers forget to include climate variability and report on climate change as if it is something happening within a short timeframe. Scientists speak about climate change as changes that take place (where) over a long period, and one needs to have records to measure the changes that took place (IPCC 2007). In the absence of records, one has to include climate variability; for example, Afful (2016) used the concept of climate variability in his conceptualisation and discussion of the extension competency in climate change. There is a need to differentiate between climate change and variability. According to WIRED UK (2018), climate change is a broad range of global phenomena created predominantly by burning fossil fuels, which add heat-trapping gases to the earth’s atmosphere. These phenomena include the increased temperature trends described by global warming, which also encompass changes such as sea-level rise; ice mass loss in Greenland, Antarctica, the Arctic and mountain glaciers worldwide; shifts in flower/plant blooming; and extreme events. Other authors view the definition from a different perspective; for example, climate change in the IPCC (2007) refers to a change in the state of the climate that can be identified by changes in the mean and/or the variability of its properties, and that persists for an extended period of time, typically decades or longer. It refers to any change in climate over time, whether caused by natural variability or as a result of human activity. This definition provides a comprehensive insight; for example, it was reported that the gradual yet dramatic disappearance of the glaciers on Mount Kilimanjaro is a result of climate change (350Africa.org). The glaciers act as a water tower and several rivers are now drying up. It is estimated that 82% of the ice that capped the mountain, when it was first recorded in 1912, is now gone (350Africa.org).

Climate has always been changing naturally; the current impact of human activities is causing the climate to change in an unnatural way and at a faster pace than ever before (Shabangu 2017). This unnatural and human-induced climate change is problematic as it is causing shifts in the normal climatic conditions such as rainfall and temperature, which, in turn, are placing pressure on the planet’s natural environment and have negative impacts on people and their livelihoods (Benhin 2006). The question that is asked is the following: what is drought and whether it is caused by climate change or not? According to the Western Cape Government (2017), there are four types of drought. The first one is meteorological drought: this is an extended period during which less than a certain amount of the normal (long-term average) rainfall is received over a large area.

The second one is hydrological drought: the impact of a reduction in rainfall on natural and artificial surface water resources. Furthermore, this type of drought occurs when there is a deficit in surface runoff below normal conditions or when there is normal depletion of groundwater supplies. Hydrological drought reduces the supply of water for irrigation and other household and industrial uses. The third one is agricultural drought: a reduction in water availability below the optimal level required by a crop during each different growth stage, resulting in impaired growth and reduced yields. Agricultural drought relates to an imbalance in water content of the soil during the growing season. The fourth one is the socio-economic drought: the impact of drought on human activities, including both indirect and direct impacts. It relates to institutional economic decision-making. Socio-economic drought occurs when demand for freshwater exceeds supply (Western Cape Government 2017).

These drought scenarios were explained in the government communication for farmers, alerting them of what to expect in the Western Cape. There was an argument as to which stage can be declared as a disaster. It is the writers’ view that all the municipalities are affected and as such the situation of drought and climate change affect all areas of the Western Cape, for example, the water rationing, especially in the city of Cape Town, is a case in point.

### Causes of climate change

Studies show that there is no single factor that causes climate change. Indications from literature are that both humans and non-humans can cause climate change (Cracknell 2011; Shabangu 2017). Humans could be engaged in activities that increase the amount of heat-trapping gases in the earth’s atmosphere, a phenomenon called ‘greenhouse gases’. According to Cracknell (2011), greenhouse gases occur naturally in the atmosphere and are important as they make the earth’s temperature warm enough for life to exist. Without these heat-trapping gases, the planet would be far too cold, making it uninhabitable.

However, as humans increase the amount of these gases in the atmosphere, more and more heat is trapped, which, in turn, is causing the climate to change. Human activities release a range of greenhouse gases; however, there are four major gases that cause climate change: carbon dioxide, methane, nitrous oxide and fluorinated gases. Carbon dioxide, according to Cracknell (2011), is the major global contributor to climate change and is released through the burning of fossil fuels (oil, coal and gas) and the removal of biomass, especially through deforestation in the tropical regions.

The second most important greenhouse gas is methane. The high emissions of methane arise from agricultural activities and practices, in particular, from the management of manure and the decomposition of organic waste. It is important to indicate that the mismanagement of manure may result in releasing more gases into the atmosphere. These gases are known to be promoting climate change.

The third key greenhouse gas is nitrous oxide, which is also released during agricultural activities, mainly through the application of nitrogen fertilisers, depending on the type of fertilisers that are being used; some fertilisers can contribute to climate change, for example, nitrogen fertilisers can decompose under anaerobic conditions to produce a gas known as nitric oxide, which can escape into the atmosphere to cause havoc, thereby destroying the cover of the atmosphere. The final category of greenhouse gas is fluorinated gases; these are emitted during industrial processes but have a minimal impact compared to other gases (Aydinalp & Cresser 2008; Cracknell 2011).

The following livestock were reported: 493 380 herd of cattle in February 2004; the Western Cape accounted for just 3.6% of the national herd, although its 2 979 410 sheep make up a more substantial 10.6%. The region also has 239 757 pigs (15.3%) and 244 915 goats (3.7%). The industry is both extensive and field-based (cattle and sheep), or intensive and based on grain feeds (poultry and pigs) (Vink & Tregurtha n.d.). Furthermore, there are 1267 milk producers in the Western Cape (Vink & Tregurtha n.d.), and the management of manure becomes important. It should be understood that no matter how little these gasses are produced by livestock, the potential to contribute to damaging the atmospheric layer serving as a cause of climate change exists in the Western Cape based on the numbers of the livestock.

### Implications of greenhouse gases to food security

Food security, according to Benhin (2006), is defined as a ‘state that prevails when people have secure access to sufficient nutritious food for normal growth, development, and an active and healthy life’. This explanation is in line with the understanding of other researchers and important institutions that promote food security (DAFF 2014). According to Cracknell (2011), greenhouse gas emissions are causing the earth to get warmer.

Warmer temperatures are causing other major changes around the world. Impacts of increased greenhouse gases in the atmosphere include a rise in weather-related incidents such as floods, droughts, frosts, hailstones and destructive storms; the extinction of countless plant and animal species; the loss of agricultural harvests in vulnerable areas; the changing of agricultural seasons; the melting of glaciers; the disruption of water supplies; the expansion of infectious tropical diseases; the rising of sea levels and much more (Benhin 2006). One of the sectors most affected by climate change is the agricultural sector as it is dependent on environmental stability in terms of water supply, atmospheric temperatures, soil fertility and the incidents of pests and disease (Benhin 2006).

The negative effects of climate change affect smallholder farmers which directly has an impact on food security. Furthermore, the people most vulnerable to climate change impacts are communities in developing countries, given their low or compromised resilience levels to climate change (Cohen et al. 2008). Communities in developing countries have limited financial and technical resources to support climate adaptation and mitigation (Benhin 2006). Smallholder farmers in rural areas, such as the tea farmers in Kenya, will be severely affected unless action is taken now to ensure that they are aware of the impacts of climate change and are supported to address these impacts using locally appropriate solutions (Cohen et al. 2008).

Climate change has significant impact on agriculture, especially on those crops that are dependent on consistent climatic conditions; hence, it affects food security (Vink & Tergurtha n.d.); it also causes the growing season to be shortened, resulting in disastrous failure (Benhin 2006). Thus, smallholder farmers who depend on agriculture may experience food shortages. It is important to note that leadership in the two departments of Local Government and the Department of Economic Opportunities responsible for Economic Development and Tourism have acknowledged and agreed that ‘extreme weather events are threatening food security and economic growth’ (Western Cape Department of Agriculture 2016:7). This paper argues that action needs to be taken to mitigate the impact of climate change in the Western Cape Province.

### Impact of climate change on crop production

Climate change and climate variability present a negative influence on crop production (Afful 2016). In areas, for example, where irrigation is insufficient, crops wither and die, thus reducing the yield. The reduced yield could further mean reduced profit and increased poverty. However, certain steps need to be taken to mitigate climate change and improve crop production. The following mitigation could be adopted. It has also been found that climate change affects crops by spreading new types of diseases that were not there in the past (Cohen et al. 2008). Such diseases and pests might be difficult to control because of a lack of registered pest control remedies.

The outbreak of the fall armyworm in South Africa, for example, presented new challenges and caused havoc to the farmers in the beginning of 2017 (M. Motupa [Limpopo Department of Agriculture, Gravelot Service Centre] pers. comm., 18 April 2017). As far as the Western Cape is concerned, the drought and heat affected fruit quality (e.g. size, sunburn, colour and storage ability) in the 2016 and 2017 season, and the demand has decreased in key markets; therefore, the export share is expected to decrease by 4.2%. A similar trend was observed in terms of vegetable production, and one tomato firm was closed down because of climate change (Western Cape Government 2017). In the Ceres region, 50% fewer onions and 80% fewer potatoes were planted because of a lack of water. The reduced production could be translated to a R40-million loss in wages to seasonal workers (Agri Western Cape, 20 September 2017) cited by the Western Cape Government (2017).

### Mitigation measures for crop production

It is very important that crops should be adapted to the agricultural environment in the wake of climate change. Research can make a contribution in the breeding of new climate-tolerant crop varieties to suit the changing climate patterns (source). Historical and current breeding practices and experience indicate that natural biodiversity within crops has allowed for plant adaptation to different conditions, providing clear evidence that plant breeding has great potential to support adaptation of crops to climate change (IPCC 1996). The development of a cropping system that can also be seen as another measure can help agriculture in the Western Cape to adapt to a changing climate (Vink 2003). It has been found that where crop mixtures were used, there has been positive results in terms of output because if several crops are growing at one time, this can help systems exhibit greater durability during periods of high water or heat stress (IPCC 1996).

According to Davies (2014), the Government of the Western Cape adopted a climate change response strategy that, among others, focussed on food security. Some of the strategies included exploring alternative crops and testing them under drought conditions, conservation farming practices, crop rotation, more efficient use of water, renewable energy farm planning, and monitoring of plant and soil changes. It is true that climate-related disasters pose significant challenges to the agricultural sector of the Western Cape. There is doubt that if this is not addressed adequately, the intensification of disaster risks associated with climate change have the potential to undermine the productivity and resilience of this sector.

The impacts also extend significantly into the wider provincial economy. It is for this reason that the Government of the Western Cape has developed a drought response strategy in which mitigation issues have been implemented. The Government of the Western Cape has rolled out a wide range of support actions in partnership with industry organisations and Agri Western Cape; for example, it provided drought support (mainly feed provision) to stock farmers, and the Avian Influenza epidemic had been well managed (Government of Western Cape 2017).

### Impact of drought on livestock production

Drought knows no size of farming; it affects both smallholder and large-scale farming. This was confirmed by Swart in 2016 who presented in the AGRA (Agricultural forum consisting of role players called to discuss drought situation) dialogue on drought, who reported that all categories of farmers were affected by climate change. There was not sufficient fodder for their livestock within the commercial farming sector, and livestock were given potato seedlings to graze on because they were not being sold because of the drought (Swart 2016). While it is argued that climate change is causing havoc in livestock, Rust and Rust (2013) observed that some of the problems in livestock cooperatives were caused by conflicts resulting from poor leadership or the development of opportunistic ideas by individual members of the cooperatives. It is important that leadership should take advantage when policies are pronounced to help the farmers. Experience showed that selfishness does not help. Farmers need to be organised to fight the scourge of drought as a team rather than by competing with each other.

Climate change will affect animal production in different ways. Rust and Rust (2013) identified four ways: changes in livestock feed, grain availability and price challenges, the impact on livestock pastures and forage crop production and quality of feed changes in livestock diseases and pests, and the direct effects of extreme weather events on animal health, growth and reproduction. Furthermore, the indirect effects of climate-driven change in animal production may result mainly from alterations in the nutritional environment. Research indicates that changes in climate, to a large extent, will affect the quality and quantity of forage (Topp & Doyle 1996).

The impact of climate change may result in the deterioration of pasture, towards lesser quality, which, in turn, will affect the quality of animals; hence, the meat quality will also be affected. Sometimes the quality of meat may be affected by infected feed that the animals eat (Rust & Rust 2013). Changes in temperature and rainfall may result in the spread of disease and parasites creeping into new regions, or an increase in the incidence of pests and diseases. Pests and diseases reduce animal productivity and increase animal mortality (Baker & Viglizzo 1998).

Other changes include heat stress which, according to Fuquay (1981), has various detrimental effects on livestock. In the dairy herd, climate change will result in a decrease in fodder production from dry land and irrigated pastures, resulting in the rise in feed costs (Rust & Rust 2013).

As far as monogastric animals are concerned, they emit gases that if not managed will go into the atmosphere and destroy the layer that protects the intense heat from destroying the environment and human beings. Poultry meat and egg production are the most efficient animal protein production systems (Rust & Rust 2013). Poultry meat production is the most environmentally efficient (smallest carbon footprint per unit product produced), followed by pork and mutton (primarily lamb) with beef the least efficient (Williams, Audsley & Sandars 2006).

Some of the emissions can be reduced by using various housing techniques that have been developed to reduce emissions. A combination of housing and feeding measures seems most promising to achieve a substantial reduction in emissions at a relatively low cost (Van der Peet-Schwering et al. 1999). As far as the Western Cape is concerned, livestock farmers are among the hardest hit by the drought. An estimated 30 000 cattle have been sold as farmers were unable to feed their herds. The impacts of the drought in the summer rainfall region on beef cattle herds, with high numbers of animals slaughtered, have been reported (Government of Western Cape 2017).

### Impact of climate change on water sources for irrigation

Climate change affects water for irrigation (Benhin 2006). It was also proved in the farmer’s perception that climate change is a reality in the Western Cape. For example, 50% of the sample interviewed by Benhin (2006) observed that the climate is becoming drier and hotter, the winter season has shortened and the rain is coming later than expected. *The Farmers Weekly* (2017) reported that in the Western Cape there was a decline in the volume of wine grapes harvested because of a lack of irrigation water (Du Preez 2017).

The dam levels have gone down to 30% (Du Preez 2017). It has been found that in other places, like some irrigation schemes such as Krokodilheuvel in the Limpopo province, irrigation has not been effective because it was a furrow system prior to the introduction of the drip and floppy systems of irrigation. It is the writers’ experience that where flood irrigation is used, it has proved to waste more water; hence, it was officially not recommended in the Limpopo province. Drip and sprinkler irrigations were recommended by the Limpopo Head Office (De Witt 2010).

It was further reported that farmers in the Western Cape have sold 30 000 cattle because of a lack of feed as a result of drought. The water availability was reported based on the rainfall received in the areas; for example, in 2016 rainfall in September was lower than 50% of the long-term mean in the northern and western parts of the Western Cape. Only the Southern Cape east of the Breede River mouth experienced normal to above-normal rainfall. In October of the same year, most areas had below-normal rainfall, with a few exceptions (Government of Western Cape 2017); this situation provides evidence that climate change is affecting the availability of water.

### Mitigation for livestock production

It is understood that mitigation is associated with reducing risk of loss from the occurrence of any undesirable event. In general, mitigation means to minimise the degree of any loss or harm (https://www.merriam-webster.com/dictionary/mitigation). According to the response policy on climate change, mitigation factors imply that steps have to be taken to lessen the impact of climate change (Davies 2014; Western Cape Department of Agriculture 2014). The Western Cape’s response strategy is a two-pronged process consisting of mitigation and adaptation. In terms of mitigation, it aims at making some contribution to national and global efforts to significantly reduce greenhouse gas emissions and build a sustainable low carbon economy in the Western Cape, while simultaneously addressing the need for economic growth, job creation and improving socio-economic conditions.

The second strategy, namely adaptation, aims to reduce climate vulnerability and develop the adaptive capacity of the Western Cape’s economy, its people, its ecosystems and its critical infrastructure in a manner that simultaneously addresses the province’s socioeconomic and environmental goals. Beef and dairy cattle can contribute to climate change through the greenhouse gasses they emit. On the contrary, beef and dairy cattle are tolerant to heat stress (Rust & Rust 2013).

However, in the case of dairy and monogastric animals, the following need to be considered: lowering the concentrations of urea and ammonia in the slurry; lowering the temperature of the slurry; reducing the emitting surface area; and reducing the pH of the slurry (Rust & Rust 2013). It is important that we need to be seen taking steps to deal with climate change and not to promote it but to adopt mitigation steps. The Western Cape will need to work and ensure that natural resources are well managed to reduce climate vulnerability. The other area to work on would be to improve resilience and coping capacity within the various communities that are also vulnerable. The other step would be to actively adapt some of the practices to climate change.

## Conclusion and recommendations

It is evident that climate change is one of the biggest challenges that humanity is facing both in the Western Cape Province and internationally. It was evident that extension advisors did not have sufficient knowledge of climate change, including its causes. The findings indicated that humans also cause climate change; hence, it is important that humans should be made aware of the concept of climate change and its impacts so that they can adopt climate smart farming techniques and practices. This research was conducted in the spirit of equipping extension advisors to take note of it in order to promote adaptation and mitigation measures.

This paper discussed the findings and specifically made reference to crops and livestock that indicate how the Western Cape has been affected by climate change, especially during the 2015 and 2016 drought. The article concluded by listing some of the mitigation steps that need to be considered in both crop and livestock production. Based on the findings, it is recommended that measures of mitigation should be adopted towards food security and these include the following:

- breeding livestock that are adaptable to the environment
- adopting precise farming strategies as pronounced in the climate change reports of the Western Cape like the policy of climate change of the Western Cape
- minimising environmental degradation like burning of organic materials because gases affect the atmospheric layer that serves as a protection against ultraviolet rays; furthermore, human beings should refrain from injudicious use of irrigation water and fertilisers; avoid overgrazing; keep the correct size of the herd; and not burn organic matter but rather adapt other practices like composting
- managing the manure of the animals and using adaptable seeds from crop breeding
- low-income people, who depend on vulnerable subsistence agriculture, should be assisted with the correct measures
- promoting climate research uptake among extension officers so that they can contribute to the body of knowledge that will mitigate or adapt to climate change
- developing more drought-resistant dry land crops and pastures for dairy consumption and adopting more effective and environmentally friendly farming practices
- conducting education and sensitisation workshops for extension advisors to enable them to fight the effects of climate change at local and regional levels.
